# Glutathione Injection Alleviates the Fluctuation of Metabolic Response under Thermal Stress in Olive Flounder, *Paralichthys olivaceus*

**DOI:** 10.3390/metabo10010003

**Published:** 2019-12-18

**Authors:** Seonghye Kim, Ahran Kim, Seohee Ma, Wonho Lee, Sujin Lee, Dahye Yoon, Do-Hyung Kim, Suhkmann Kim

**Affiliations:** 1 Department of Chemistry, Center for Proteome Biophysics, and Chemistry Institute for Functional Materials, Pusan National University, Busan 46241, Korea; seonghyeee@pusan.ac.kr (S.K.); ahran110@naver.com (A.K.); metabomsh@pusan.ac.kr (S.M.); wonholee@pusan.ac.kr (W.L.); isujin@pusan.ac.kr (S.L.); dahyeyoon@korea.kr (D.Y.); 2 Department of Aquatic Life Medicine, Pukyong National University, Busan 48513, Korea; dhkim@pknu.ac.kr; 3 Department of Herbal Crop Research, National Institute of Horticultural and Herbal Science, RDA, Eumseong 27709, Korea

**Keywords:** glutathione injection, thermal stress, metabolomics, NMR, olive flounder

## Abstract

Continuous increases in water temperature disturb homeostasis and increase oxidative stress in fish. Glutathione (GSH) is an intracellular antioxidant that helps to relieve stress in animals. In this study, we observed the effect of GSH on olive flounder exposed to high temperature using serum parameters and NMR-based metabolomics. Based on the results from the first experiment, 20 mg of GSH was chosen as an effective dose with lower infection rates and mortality. Then, fish were divided into Control, Temp (PS injection), and GSH (glutathione injection) groups, and fish in Temp and GSH groups were exposed to temperature fluctuations (20 °C→24 °C→27 °C). In OPLS-DA score plots, Temp group was clearly distinguished from the other groups in the kidney. In the liver, the metabolic patterns of GSH group were close to the Temp group on day 4 and became similar to Control group from day 7. Serum parameters did not change significantly, but the deviation in Temp group was greater than that in GSH group. Metabolite levels that were significantly altered included GSH, lactate, O-phosphocholine, and betaine in the kidney and taurine, glucose, and several amino acids in the liver, which were related to antioxidant activity and energy system. Therefore, GSH supplements could relieve thermal stress influencing metabolic mechanisms in fish.

## 1. Introduction

Environmental stressors are detrimental to fish when they are present at concentrations above the normal range. A stressed fish undergoes physiological and biochemical changes to maintain homeostasis and attempts to return to prestress conditions to overcome the stress [1]. Fish are poikilothermic (ectothermic) animals and cannot avoid alternations, such as changes in salinity and temperature and exposure to pollution, in their surrounding environment [2]. Water temperature is important to the well-being and survival of fish [3]. Global climate change is rapidly worsening and one ongoing effect is an increase in the ocean temperatures. According to the Ministry of Oceans and Fisheries, the average water temperature of Korea’s coast has risen by 2.89 °C over the past decade, and the coastal water temperature in July 2018 reached over 28.5 °C on Jeju Island, which has a lot of aquaculture farms of olive flounder. Olive flounder (*Paralichthys olivaceus*), one of the major fish species in the aquaculture industry of South Korea, is a stenothermal fish that survives at an optimal temperature of 20 °C. In fish, a 10 °C increase in water temperature will double the rate of physiological function and significantly increase mortality [4]. Most studies have conducted thermal stress experiments at 30–34 °C, which are excessively high temperatures and cannot reflect the effects in the real environment [5,6,7].

Changes in water temperature beyond the optimal range affect the functioning of fish. Such changes lead to the secretion of cortisol, a primary stress hormone, and to fluctuations in energy metabolism and osmoregulation because of temperature-influenced enzyme traits [8]. In addition, high water temperature causes increases in the generation of reactive oxygen species (ROS) in fish. The overproduction of ROS can damage cellular components, such as cell membranes, DNA, and protein, and can induce oxidative stress [9,10,11]. Oxidative stress can cause severe consequences from the modification of cellular functions to death. To alleviate oxidative stress, cells possess antioxidant components, including glutathione (glutamyl-cysteinyl-glycine; GSH). GSH works mainly as an antioxidant defense mechanism and is involved in nutrient metabolism, protein synthesis, and the regulation of other cellular events in human and animals [12]. It was confirmed that the inoculation of the vaccine with GSH was effective for the reduction of stress and inflammatory response in calves [13]. And, the effect of GSH in the antioxidant system has been investigated in environmental and chemically stressed fish [14], and the influence of increased temperature on the levels of GSH in multiple tissues has been reported [15]. GSH is mainly given through oral administration to fish in aquaculture farms, but the injection can be a more effective way to administer GSH, because this requires a smaller amount of materials in one treatment, and the duration is longer than the oral route. The injection of GSH into rainbow trout (*Oncorhynchus mykiss*) rapidly increased GSH levels in the liver, kidney, and gills without the inhibition of additional GSH synthesis, unlike the case in mammalian tissues, and it could serve as an antioxidant [16,17]. The GSH system in fish is flexible and can rapidly react to high GSH demands under stressful conditions.

In general, the monitoring of hematological parameters such as plasma cortisol and metabolic enzyme activities are used to detect stress in fish [18,19,20]. However, these detectable physiological responses cannot always be elicited as stress responses, and the lack of these factors does not always mean that the fish are stressed. Also, they must be complemented with other measurements due to their high variability and they are related to metabolic changes as an adaptation or acclimation mechanism [21]. In previous studies, metabolomics analysis identified a decrease of amino acids including glutamine, tyrosine, and phenylalanine) and alternation in lipid metabolism in high thermal-stressed Atlantic salmon (*Salmo salar*) [18]. Further, the disturbance of the energy metabolism, including decreased ATP, phosphocreatine, and glycogen levels, has been observed in rainbow trout (*O. mykiss*) under high temperatures [22]. Using blood parameters, metabolomics can better explain the physiological changes in stressed fish [23]. The metabolomics approach using nuclear magnetic resonance (NMR) enables the determination of the levels of small metabolites with high reproducibility and fast screening along with the evaluation of the ongoing metabolic alteration. The metabolites in intact tissues can be measured using high-resolution magic angle spinning (HR-MAS) NMR without any extraction process of the metabolites and the possibility of experimental loss. In the present study, the appropriate concentration of GSH injection for *P. olivaceus* was determined. The changes of metabolites in the kidney and liver of *P. olivaceus* was investigated, and the physiological responses under thermal-stress were confirmed with changes in the hematological parameters and NMR-based metabolomics. The objectives of this study are to elucidate the effect of GSH on stressed fish during an increase in temperature as well as when the fish were maintained at a high temperature.

## 2. Results

### 2.1. Glutathione Injection Test

**Figure 1:**
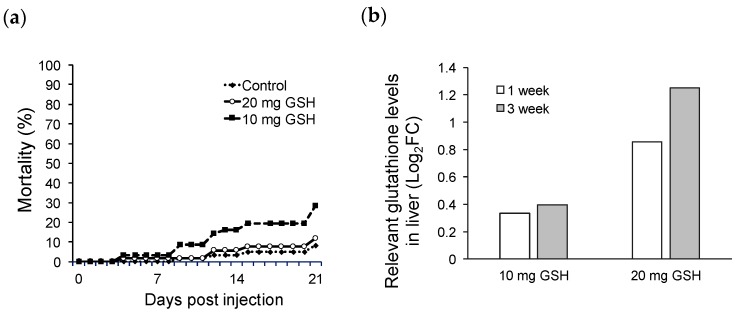
Mortality rate (**a**) of the glutathione injection experiment and (**b**) concentrations of glutathione in the liver (**a**) PS injection (Control), 10 mg of GSH injection (10 mg GSH), and 20 mg of GSH injection (20 mg GSH). (**b**) The relevant GSH levels (log_2_(fold change)) in the 10 mg and 20 mg GSH groups compared to the Control group at 0 week were 0.35 and 0.85 at 1 week, and 0.40 and 1.25 at 3 weeks, respectively.

In the first experiment, natural infection by *Edwardsiella piscicida* was observed from the first week and over a 40% infection rate was observed until the third week in the Control and 10 mg GSH groups. The 20 mg GSH group showed the lowest infection rate for the experimental period (Table 1). The Control showed the lowest mortality of 8.33%, but the highest infection rate of 60% at three weeks. This indicates that although fish did not lead to mortality during the observation period, they could cause a potential mortality because they retained bacteria. And, the final mortality rate was 8.33%, 11.83%, and 28.50% in the Control, 20 mg GSH, and 10 mg GSH groups, respectively, indicating that the injection of 20 mg of GSH was more effective than that of 10 mg of GSH (Figure 1a). The concentration of GSH in liver significantly increased in the injection group compared to the Control group and was maintained for three weeks (Figure 1b). Based on these results, the second experiment was performed using an injection of 20 mg of GSH.

### 2.2. Effect of Glutathione on Thermal Stress

No mortality and infection occurred during the experiment. The variable of this experiment was only a temperature increase, similar to that seen in the environment

#### 2.2.1. Results of Serum Parameters

The differences in serum biochemical parameters among the groups are shown in Figure S1. The blood urea nitrogen (BUN), total protein (TP), and total cholesterol (TCHO) were significantly changed (*p* < 0.05) in the Temp group at day 10. Alanine aminotransferase (ALT) and aspartate aminotransferase (AST) in the Temp group showed dramatic increases on days 4 and 10, respectively. Compared to the Temp group, the GSH group had a smaller variation in the measured parameters.

#### 2.2.2. Metabolic Changes in Kidney and Liver under Thermal Stress

**Figure 2:**
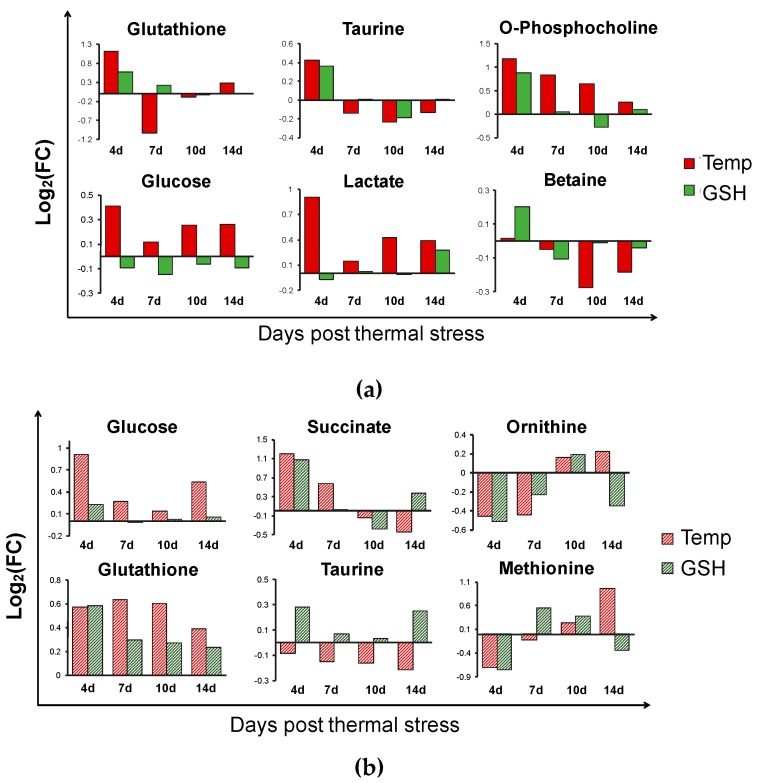
Relative changes in metabolites of the kidney (**a**) and liver (**b**) exposed to thermal stress. Values in Temp and GSH groups are shown as log_2_(fold change) relative to the Control group at each sampling time point. d, day; Group (Temp group, Temp; GSH group, GSH); PC, O-Phosphocholine.

In the present study, metabolite analysis based on NMR was conducted, and a total of 37 metabolites were assigned in both the kidney and liver. The normalized concentrations and fold changes (FC) of all metabolites are shown in Supplementary Tables S1 and S2, respectively. In the kidney, most metabolites changed significantly in the Temp group on day 4. GSH, O-phosphocholine (PC), taurine, glucose, and lactate were significantly increased in Temp group (Figure 2a). Further, betaine and myo-inositol were decreased after day 7. In the liver, GSH, taurine, methionine, glucose and amino acids were identified as significant metabolites (Figure 2b). According to the pathway analysis, the results showed that 16 pathways were identified (*p* < 0.05) (Table 2). The most significant pathways were amino acid metabolism including alanine, aspartate and glutamate metabolism; glycine, serine and threonine metabolism and valine, leucine and isoleucine biosynthesis.

**Figure 3:**
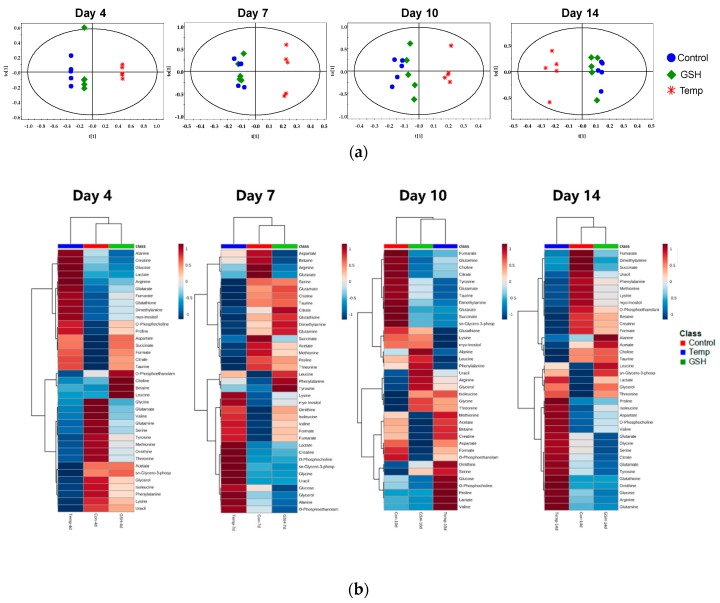
OPLS-DA score plots (**a**) and heat maps (**b**) of the kidney. (**a**) OPLS-DA score plots of the ^1^H NMR spectra of the kidney sample for each time point. Dot and color represent sample and group, respectively. Day 4 (Q^2^ = 0.611), Day 7 (Q^2^ = 0.318), Day 10 (Q^2^ = 0.191), and Day 14 (Q^2^ = 0.135) (*n* = 5/group). Red asterisk (Temp group) is separated from the Control and GSH groups. (**b**) For heat maps, each colored cell corresponds to a concentration value, with groups in rows and metabolites in columns. In the dendrogram, Control and GSH groups are closer than Temp group except for Day 10.

**Figure 4:**
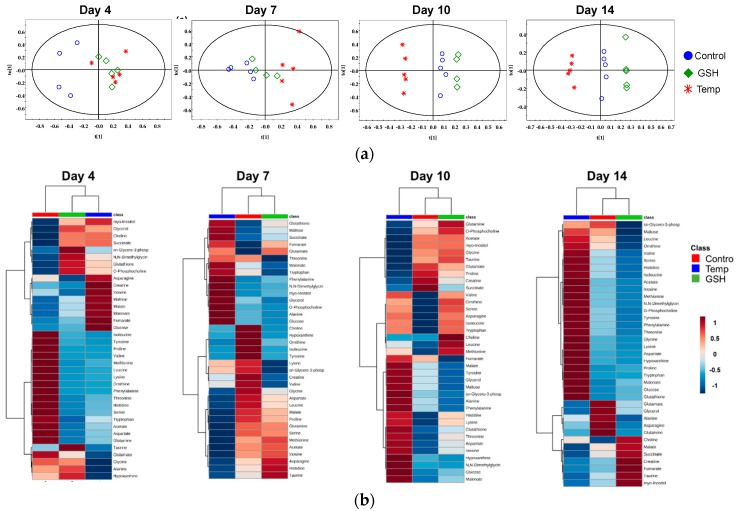
OPLS-DA score plots (**a**) and heat maps (**b**) of the liver. (**a**) OPLS-DA score plots of the ^1^H NMR spectra of the liver sample for each time point. Dot and color represent sample and group, respectively. At days 4, 7, and 10, one sample was removed as the outlier; Day 4 (Q^2^ = 0.209), Day 7 (Q^2^ = 0.531), Day 10 (Q^2^ = 0.295), and Day 14 (Q^2^ = 0.534, *n* = 5). The green diamond (GSH group) are closer to the Control group except for day 4. (**b**) For heat maps, each colored cell corresponds to a concentration value, with groups in rows and metabolites in columns. In the dendrogram, Control, and GSH groups are closer, except for on day 4.

Heat maps visualized the metabolic changes of the Temp and GSH groups exposed to water temperature fluctuation (Figure 3b and Figure 4b). Each column displays the average metabolite concentration in each group. Similar trends of metabolic changes in the Control and GSH groups were observed in both organs as the temperature increased. In the kidney, similar patterns were observed in the Control and GSH groups, except for on day 10. The pattern of the GSH group was similar to that of the Control group or located in the middle of the two groups. In the liver, the metabolic pattern in the heat maps of GSH and Temp groups differed from that of the Control group on day 4. However, the metabolites of the GSH group changed, similar to what was observed in the Control group on days 14.

#### 2.2.3. Results of Multivariate Data Analysis

Orthogonal partial least squares discriminant analysis (OPLS-DA) score scatter plots of the NMR spectra were conducted to discriminate the metabolic patterns and are shown in Figure 3a and Figure 4a. In the scores plot, each point represents a single spectrum. The results showed different effects of thermal stress depending on the organs. In the kidney, the Temp group were significantly different from the Control and GSH groups with a reasonable Q^2^ value, and the distance between the Control and Temp groups was the largest on day 4 (Figure 3a). The GSH group was clustered with the Control group, whereas the Temp group was on the opposite side until day 14. In the liver, the separation between the Control and the experimental groups was observed on day 4 (Figure 4a). After that, the GSH group showed changes closer to those observed in the Control group. On day 14, the Temp group was significantly discriminated in the score plot.

## 3. Discussion

The injection of 20 mg of GSH increased the concentration of GSH in the liver and was effective for survival and defense against the infection. Therefore, exogenous GSH can remain functional in fish tissues. This may affect immune responses via the modulation of T-helper cell, IL-2, antibody production, and expression of immune-related genes [24]. Our results also showed better survival rates and resistance to natural infection with bacteria in a dose-dependent manner.

The activities of AST, ALT, and alkaline phosphatase (ALP) were used as indicators of liver damage in fish [19]. The emission of ALT and AST may be caused by liver damage under high temperatures and they increased in Temp group. Similar results were found in the blunt snout bream (*Megalobrama amblycephala*) with up-regulation of apoptosis-related gene expression in the liver at 35 °C [25]. The results obtained for the blood parameters fluctuated because of the high temperature and the deviation in the Temp group was greater than that in the GSH group. Therefore, GSH might help reduce stress-induced fluctuation of biochemical parameters in fish serum by thermal changes.

From the multivariate analysis results, it was determined that the thermal stress affected the variation of the metabolites in the kidney. In the OPLS-DA score plot, the Temp group was always separated from the GSH and Control groups. The well-defined clusters in the heat maps show that the metabolites of the Temp group were altered in an opposite manner to those of the Control and GSH groups. Additionally, the number of significant metabolites with FC < 0.5 or FC > 1.5 in the Temp group was the highest on day 4 (Table S1). Therefore, GSH injection reduced the fluctuations caused by high temperatures in the kidney from the first heating stage. On day 4, the GSH level in the Temp group was greatly increased along with taurine, in which FC was over 2.0 and decreased expect on days 7 and 10 (Figure 2a). However, in the GSH group, they maintained levels similar to the Control group. These metabolites function as intercellular antioxidants that protect the tissue from oxidative stress. In a previous study [16], GSH level in the kidney was similar to, or more than, that in the liver and it was reported that the GSH injection increased GSH levels the most in the kidney, followed by the liver. This suggests that the kidney of fish is involved in antioxidant protection, similar to the liver. An increase in water temperature led to intercellular metabolic activation producing ROS [16]. GSH in the kidney of the spotted snakehead (*Channa punctata*) decreased at 32 °C and it was maintained at a higher level after transfer to 20 °C [26]. However, in the present study, GSH significantly and continuously decreased due to the maintenance of a high temperature. Together with GSH, taurine is a major intracellular antioxidant and regulates the inflammatory response [27,28]. It has been reported that dietary taurine supplementation can reduce oxidative stress and the disturbance of Ca^2+^ homeostasis induced by thermal stress in pufferfish (*Takifugu obscurus*) [29]. After day 4, taurine continuously decreased in the Temp group. It also decreased in the GSH group on day 10, but the GSH levels recovered on day 14 similarly to the Control group. Therefore, heat stress induced decreases in GSH and taurine, and it may be the result of a disorder in the antioxidant defense system of the kidney.

Despite the high glucose levels, the lactate levels in the Temp group also increased significantly on days 4 and 14 (Figure 2a). In the GSH group, the lactate level did not change compared to the Control group, except on day 14. Exposure to acute heat stress can induce the reduction of oxygen in organisms [30]. Lactate is the main product of anaerobic metabolism. The levels of lactate typically increase in stressed fish and lactate was reported to be a potential indicator of heat resistance in the olive flounder [31]. In addition, lactate production has been known to increase to satisfy the energy requirements in turbot (*Scophthalmus maximus)* [31], sockeye salmon (*O. nerka*) [32], and killifish (*Fundulus heteroclitus*) [8] after thermal stress. The results in the present study indicate that the energy metabolism in the kidney was influenced at higher temperatures and the GSH group was less affected.

Additionally, PC and sn-Glycero-3-phosphocholine (GPC) in glycerophospholipid metabolism are significantly changed in kidney. The levels of PC continuously increased in the Temp group, and GPC increased on days 7 and 14 with an FC of over 1.2. PC and GPC are major components of membrane lipids. The increase in PC under stressful conditions may be associated with a collapse of phosphatidylcholine, which is a structural element in the cell membrane [33,34]. It is possible that thermal-induced oxidative stress caused cell disruption and resulted in the release of these metabolites. Besides, the kidney is the organ that controls the osmoregulatory function in teleost fish. The hormone hypersecretion induced by high temperatures can cause mineral metabolic changes, which can affect osmoregulation in fish [35]. After day 10, betaine and myo-inositol significantly decreased in the Temp group (Figure 2a). Although not significant, an increase of betaine in the GSH group was observed on day 4. Betaine is an effective osmoprotectant and functions by accumulating in cells. In addition, it exerts many effects as a methyl group donor for protein synthesis and other metabolically vital substances [36]. Myo-inositol levels and the activation of enzymes related to betaine synthesis were increased to maintain osmolality allostasis in tilapia when exposed to salinity stress [37,38]. Therefore, the decrease in these metabolites likely means that high water temperature leads to the impediment of osmosis mechanisms in the kidney. This result is similar to the osmotic and ionic disturbances reported in handling stressed juvenile Senegalese sole (*Solea senegalensis*) [39].

Unlike in the kidney, the liver was markedly affected on day 4 by thermal stress and GSH injection can alleviate the effect of thermal stress after the second heating stage in the liver. The liver is the main organ that regulates energy consumption. Energy and oxygen consumption are increased to account for the cost of the stress response under thermal stress exposure [40]. In addition, the stress-induced antioxidant defense and homeostasis disturbance are high-energy-demanding processes. Cortisol is a corticosteroid and is secreted as a primary response to thermal stress in rock goby (*Gobius paganellus*) [5], coho salmon (*O. kisutch*) [41], and seabass (*Dicentrarchus labrax*) [42]. Cortisol stimulates energy metabolism and the breakdown of glycogen to glucose in the liver [43]. In the present study, glucose significantly increased in the Temp group, except on day 10, and there was no marked change in the GSH group (Figure 2b). A previous study showed similar results, where an increase in glucose was observed in the liver of the Patagonian blenny (*Eleginops maclovinus*) at 14 °C, which is 4 °C higher than the optimal temperature, with decreased glycogen and increased activity of enzymes related to carbohydrate metabolism [8]. On day 4, glucose and succinate significantly increased with significant decreases in aspartate, histidine, isoleucine, leucine, lysine, proline, tyrosine, phenylalanine, and valine in the Temp and GSH groups (Table S2). When exposed to stress, amino acids are converted into the citric acid cycle substrates for energy production [44,45]. In the present study, the decreased tyrosine and phenylalanine could be the result of conversion to fumarate. These decreases of amino acids suggest that the energy demands increased and the supplement for energy production was heightened. In addition, glyoxylate and dicarboxylate metabolism was significant in this study, and this participates in energy metabolism as a bypass to the TCA cycle, and plays an important role in many fungi [46]. Additionally, maltose significantly increased in the Temp and GSH groups, except for on day 14. Maltose could be involved in energy replenishment by its degradation into two molecules of glucose. A similar reestablishment of energy metabolism at high temperatures, as has been reported previously in the Atlantic salmon (*Salmo salar*) [18]. Glucose also increased on day 14. However, the changes in amino acids were the opposite of those seen on day 4. This may be the result of protein degradation because of the high temperature [47]. Protein degradation produces ammonia, and the ornithine-urea cycle converts ammonia into urea primarily in the liver [48]. A significant decrease of ornithine on days 4 and 7 was observed in both experimental groups, which could be the result of ammonia detoxification. In the heat maps of days 10 and 14, these amino acids are indicated by a red color in the Temp group relative to the Control and GSH groups which are blue. This suggests that thermal-induced protein damage could be alleviated by GSH supplementation.

**Figure 5:**
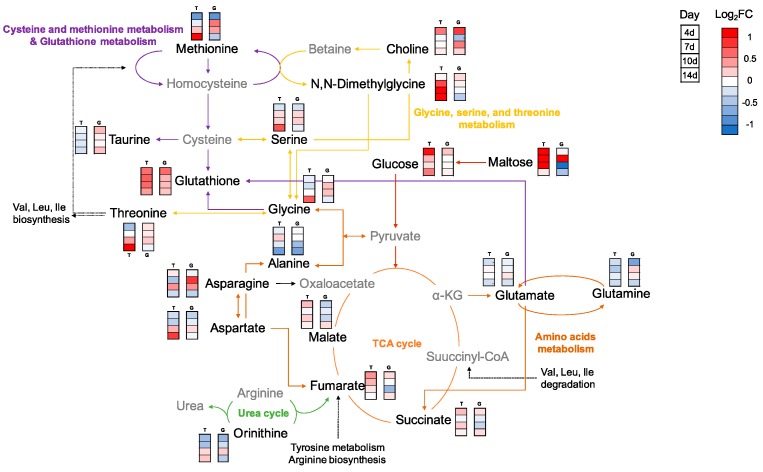
Schematic figure of metabolic pathways in liver. The red and blue colors in the bar show the increase and decrease in relative concentrations of metabolites compared to the Control, respectively. The orders from up to down represent day post thermal stress. GSH increased in both groups as temperature increased. Grey colored metabolites were not assigned in this study. T, Temp group; G, GSH group; d, day; FC, fold change; α-KG, α-ketoglutarate.

Compared to the Control group, GSH increased in both groups during the experiment. The elevated GSH levels in the GSH group could be the result of thermal stress and supplementation, consistent with our results in Figure 1b. However, the GSH levels increased more in the Temp group than in the GSH group even though they were placed under the same stressful conditions. The taurine levels increased in the GSH group but decreased in the Temp group relative to the Control group (Figure 2b). After day 4, the taurine level in the GSH group did not change compared to the Control group, but significant increases were observed on day 14 with decreases in GSH. In addition, GSH and taurine decreased in the Temp group on day 14. This suggests that thermal stress at temperatures higher than 27 °C may increase oxidative stress and affect liver antioxidant mechanisms. Methionine, glycine, and glutamate decreased in the Temp group but did not significantly change in the GSH group. Methionine is trans-sulfurated to cysteine, which is the main precursor of antioxidants such as GSH and taurine (Figure 5) [49]. The decreased methionine could be used to synthesize GSH or taurine. High temperatures may induce intracellular oxidative stress and stressed fish need to activate GSH synthesis. In the Temp group, methionine was more biased to GSH synthesis than taurine in the Temp group and this lack of taurine continued to day 14. However, in the GSH group, taurine was maintained at a level higher than or similar to that in the Control group and it could support the anti-oxidative effect [35]. This result suggests that thermal stress causes excessive synthesis of GSH in the liver as a defense against oxidative stress and GSH supplement could maintain homeostasis of antioxidants.

To the best of our knowledge, this is the first study to evaluate the effects of GSH on thermal-stressed fish using combined analysis with NMR-based metabolomics and serum biochemical tests. The 20 mg GSH injection was effective in lowering the infection rate and mortality. After the fish were exposed to thermal stress, metabolic profiling was conducted and significant changes due to high temperatures and GSH injection were identified by comparison with the Control group. The results showed that high temperature induced dynamic metabolic changes and the stress response to heat was different depending on the organ. The fluctuations in metabolite changes and hematological parameters were smaller in the GSH group than in the Temp group. NMR-based metabolomics is a powerful approach to elucidate metabolic changes as a response to stress and it can detect stress even if the physiological response does not change significantly. These findings confirmed the efficacy of GSH in improving homeostasis and alleviating the effects of heat stress. Because fish are exposed to multiple stressors, further studies are required to validate that GSH is efficacious for various stresses in fish and not only for heat stress as in the present study.

## 4. Materials and Methods

### 4.1. Glutathione Injection Test

All animal experimental procedures were carried out in accordance with the guidelines and regulations and with the ethical approval from the Ethics Committee of Pukyong National University (approval number: 2017–2010).

The first experiment was designed to determine the effective dose and duration of GSH. Healthy olive flounder (average body weight (BW) = 35 g) were obtained from a commercial fish farm in South Korea and the fish were acclimatized in a 1000-L flow-through tank at the Institute of Fisheries Science, Pukyong National University before the commencement of experiments under rearing conditions in aerated and circulated seawater at 20 °C. A total of 180 fish were randomly split into six tanks (30 fish in 250-L flow-through tanks). A GSH (reduced glutathione) suspension for inoculation was prepared by dilution in sterile physiological saline (PS). Intramuscular injections were performed with 100 µL of PS into the fish in tank 1 (Control), 100 µL of 10 mg/kg BW GSH into the fish in tank 2 (10 mg GSH), and 20 mg/kg BW GSH into the fish in tank 3 with 100 µL (20 mg GSH). Fish were monitored for 3 weeks and the mortality and infection rate were measured at 0, 1, and 3 weeks. Five fish were sampled from each group to investigate the infection rate and GSH concentration in the liver. GSH concentration was measured using NMR. Bacterial infection was observed by stamping the fish kidney on brain heart infusion agar (Difco) supplemented with 1% NaCl and incubated at 28 °C for 24 h.

### 4.2. Thermal Stress Exposure

**Figure 6:**
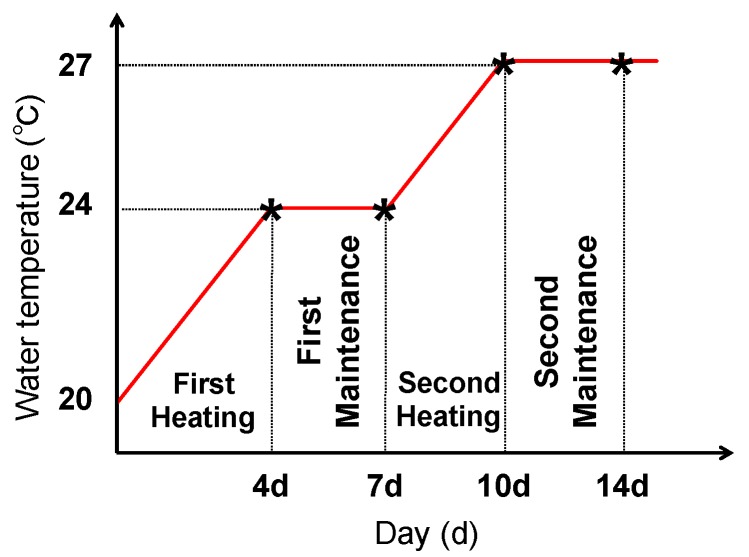
Scheme of the thermal stress experiment. Red line and asterisk represent water temperature changes and sampling time points for experiment, respectively.

For the second experiment, 165 fish with an average BW of 30 g were obtained and divided into three groups (*n = 55* in a tank): No change in water temperature after injection with PS (Control group); change in water temperature after injection with PS (Temp group); and change in water temperature after injection with GSH (GSH group). A total of 200 µL of PS was injected into the Control and Temp groups and the same volume of 20 mg/kg BW GSH into the fish of the GSH group. In the Temp and GSH groups, the water temperature was gradually increased by 1 °C each day for 4 days after injection at 20 °C (day 4, first heating). After keeping at a constant 24 °C for 3 days (day 7, first maintenance), the temperature was increased to 27 °C (day 10, second heating) and maintained for 3 days at 27 °C (day 14, second maintenance) (Figure 6). The Control group was maintained at 20 °C over the experimental period. The experiment lasted for 2 weeks and the fish were not fed to minimize the stress caused by water contamination. Five fish were sampled randomly from each tank at days 4, 7, 10, and 14, and euthanized using MS-222. Blood was taken from the caudal vein and allowed to stand for 15 min at room temperature (20–25°C) followed by 4 °C overnight, and the serum was isolated after centrifugation at 6500 rpm for 5 min at 4 °C. The kidney and liver samples were removed aseptically, and all samples were stored at −80 °C until subsequent use.

### 4.3. Analysis of Biochemical Parameters in Serum

Blood samples were taken from each group at days 0, 4, 7, 10, and 14. For biochemical analysis in serum, AST, ALT, BUN, TCHO, TP, and ALP were measured using a FUJI DRI-CHEM 4000i (FUJI PHOTO FILM Co., Tokyo, Japan). Statistical analyses were conducted with one-way analysis of variance for multiple comparisons (ANOVA).

### 4.4. ^1^H NMR Measurement

For HR-MAS NMR measurements, 20 mg of an intact kidney sample (*n = 5*) was weighed and carefully placed into a 4 mm NMR nanotube. A total of 20 µL of phosphate buffer (pH 7.4) in deuterated water (D_2_O) containing 2 mM 3-trimethylsilyl-2,2,3,3-tetradeuteropropionicacid-d_4_ (TSP-d_4_, Sigma-Aldrich, St. Louis, MO, USA) was added as an internal chemical shift standard. The liver is inappropriate for HR-MAS because it contains lots of lipids and the peaks of lipids are broad with a wide range. Therefore, polar metabolites were extracted. The lyophilized liver samples were homogenized with a mortar and pestle. The homogenized samples were extracted with acetonitrile/water (1:1, v/v) [50]. The supernatant was collected after centrifugation (3000 rpm, 4 °C, 10 min) and freeze-dried. Completely dried samples were reconstituted with 700 µL of D_2_O containing 2 mM TSP-d_4_ and transferred into a 5 mm NMR tube. ^1^H-NMR spectra of the intact kidney tissues were measured using a 600 MHz HR-MAS NMR spectrometer with a nano-NMR probe (Agilent Technologies, Santa Clara, CA, USA). The spinning rate was 2050 Hz with a degree of magic-angle (54.74°) at a temperature of 24.85 °C (298 K). The spectra of the liver extracts were acquired using a liquid probe. A Carr-Purcell-Meiboom-Gill pulse (CPMG) sequence was used to suppress the large peaks of high molecular mass compounds. Spectra were acquired using an acquisition time of 1.703 s, relaxation delay of 1 s, and 128 transients. The acquired spectra were phased, and the baseline was corrected and referenced to the TSP-d_4_ peak (chemical shift of 0 ppm) using VnmrJ 4.2 software (Aligent Technologies, Santa Clara, CA, USA).

### 4.5. Statistical Analysis of ^1^H NMR Data

All spectra were analyzed using the Chenomx NMR Suite 7.1 Professional (Chenomx Inc., Edmonton, AB, Canada) for the identification and quantification of metabolites based on a 600 MHz NMR library database. The concentrations of metabolites were normalized by the sum of total concentration to reduce sample-to-sample variation. The statistical significance of metabolites was determined using a *t*-test (two-tailed, equal variances, *p* < 0.05). The FC of each metabolite was calculated as being divided by the average of the Control group. Multivariate statistical analysis was performed to confirm the distinction in the metabolic patterns between the Control and thermal-stressed groups. The region of the spectra between 0.5 and 9.0 ppm was binned with a binning size of 0.001 ppm. The areas of residual water and sideband were removed. The binned data of each NMR spectrum were normalized with Pareto scaling and imported into the OPLS-DA model using SIMCA P+ (Umetrics, Sweden). The horizontal component of the scattered OPLS-DA score plots indicates the variation between the group. The Q^2^ parameter was used to evaluate the predictability. For an overview of the metabolites among groups, a heat map of the metabolites concentrations was performed using MetaboAnalyst 4.0. The Kyoto Encyclopedia of Genes and Genomes (KEGG) pathway analysis for the assigned metabolites was performed using, MetaboAnalyst 4.0 with a threshold limit of *p* < 0.05.

## Figures and Tables

**Table 1:** Infection rate of olive flounder in the glutathione injection test.

Week	Control (%)	20 mg GSH (%)	10 mg GSH (%)
0	0/5 (0)		
1	1/5 (20)	1/5 (20)	2/5 (40)
3	3/5 (60)	0/5 (0)	2/5 (40)

%: Percentage of infection.

**Table 2:** Top significant metabolic pathways from the MetaboAnalysis.

No.	Pathway Name	Hits	Total	*p*-Value
	**Amino acid metabolism**			
1	Alanine, aspartate and glutamate metabolism	8	28	6.41 × 10^−7^
2	Arginine biosynthesis	6	14	1.23 × 10^−6^
3	Glycine, serine and threonine metabolism	7	33	3.03 × 10^−5^
4	Valine, leucine and isoleucine biosynthesis	8	4	4.37 × 10^−5^
5	Arginine and proline metabolism	48	5	0.004448
6	Phenylalanine, tyrosine and tryptophan biosynthesis	4	2	0.0049789
7	Histidine metabolism	16	3	0.010452
8	Phenylalanine metabolism	10	2	0.043482
	**Metabolism of other amino acids**			
9	Glutathione metabolism	28	4	0.0082529
10	D-Glutamine and D-glutamate metabolism	6	2	0.011982
11	beta-Alanine metabolism	21	3	0.022376
	**Carbohydrate metabolism**			
12	Glyoxylate and dicarboxylate metabolism	32	8	1.98 × 10^−6^
13	Citrate cycle (TCA cycle)	20	4	0.0023246
14	Pyruvate metabolism	22	4	0.0033598
15	Galactose metabolism	27	3	0.043482
	**Lipid metabolism**			
16	Glycerophospholipid metabolism	36	4	0.019962

## Supplementary Materials

The following are available online at https://www.mdpi.com/2218-1989/10/1/3/s1, Table S1: Normalized concentration of metabolites in the kidney, Table S2: Normalized concentration of metabolites in the liver, Figure S1: Changes in the biochemical parameters of serum in olive flounder after thermal stress.
