# Supplementary material for: Glutathione Injection Alleviates the Fluctuation of Metabolic Response under Thermal Stress in Olive Flounder, Paralichthys olivaceus
Source: Metabolites. 2019 Dec 18;10(1):3. doi: 10.3390/metabo10010003 (PMC7022829; doi:10.3390/metabo10010003)

Table S1. Normalized concentration of metabolites in the kidney.

Normalized concentrations are expressed as means ± standard deviation (SD).

Fold change = (concentration of treatment)/(concentration of control)

*, P < 0.05; **, P < 0.01; ***, P < 0.001

Day 4	Control – 4d			Temp – 4d				GSH – 4d			
Metabolites	Mean	±	SD	Mean	±	SD	FC	Mean	±	SD	FC
Acetate	0.55	±	0.05	0.49	±	0.07	0.90	0.54	±	0.06	1.00
Alanine	1.81	±	0.09	2.01	±	0.29	1.11	1.71	±	0.12	1.06
Arginine	1.56	±	0.32	2.70	±	0.28	1.73***	1.75	±	0.95	0.89
Aspartate	2.25	±	0.20	2.36	±	0.22	1.05	2.37	±	0.21	0.95
Betaine	0.75	±	0.11	0.75	±	0.16	1.01	0.86	±	0.45	0.87
Choline	0.98	±	0.12	0.94	±	0.11	0.96	1.24	±	0.10	0.79**
Citrate	0.29	±	0.05	0.41	±	0.06	1.43**	0.39	±	0.21	0.74
Creatine	1.05	±	0.21	1.48	±	0.13	1.41**	0.90	±	0.11	1.18
Dimethylamine	0.13	±	0.02	0.15	±	0.01	1.17*	0.13	±	0.02	0.94
Formate	0.14	±	0.02	0.18	±	0.03	1.25*	0.18	±	0.07	0.82
Fumarate	0.16	±	0.06	0.29	±	0.03	1.78**	0.22	±	0.10	0.74
Glucose	1.57	±	0.24	2.09	±	0.29	1.33*	1.47	±	0.33	1.07
Glutamate	9.37	±	0.74	8.56	±	0.28	0.91*	8.71	±	1.47	1.08
Glutamine	3.61	±	0.47	3.10	±	0.25	0.86*	3.29	±	0.24	1.10
Glutarate	0.46	±	0.12	0.58	±	0.05	1.26	0.50	±	0.18	0.92
Glutathione	0.38	±	0.09	0.83	±	0.12	2.16***	0.57	±	0.14	0.67*
Glycerol	1.55	±	0.38	1.20	±	0.12	0.78	1.42	±	0.71	1.09
Glycine	7.25	±	1.19	4.95	±	0.41	0.68**	5.14	±	0.17	1.41**
Isoleucine	2.41	±	0.24	1.12	±	0.10	0.47***	1.88	±	1.25	1.28

Lactate	1.36 ± 0.30	2.55 ± 0.72	1.88**	1.30 ± 0.12	1.05
Leucine	2.74 ± 0.37	2.72 ± 0.28	0.99	3.74 ± 0.33	0.73**
Lysine	4.08 ± 0.19	2.50 ± 0.16	0.61***	3.62 ± 0.71	1.13
Methionine	0.97 ± 0.15	0.53 ± 0.12	0.54***	0.74 ± 0.38	1.33
Myo-Inositol	2.89 ± 0.29	5.34 ± 0.61	1.85***	3.87 ± 1.19	0.75
O-Phosphocholine	0.12 ± 0.05	0.28 ± 0.09	2.28**	0.22 ± 0.08	0.54*
O-Phosphoethanolamine	1.57 ± 0.18	2.01 ± 0.35	1.29*	1.78 ± 0.26	0.88
Ornithine	2.28 ± 0.44	1.69 ± 0.28	0.74*	1.95 ± 0.51	1.17
Phenylalanine	2.04 ± 0.40	1.58 ± 0.09	0.77*	1.84 ± 0.23	1.11
Proline	1.81 ± 0.27	2.00 ± 0.58	1.10	1.93 ± 0.18	0.94
Serine	7.89 ± 0.43	6.53 ± 0.25	0.83***	6.95 ± 0.44	1.14**
sn-Glycero-3-phosphocholine	0.75 ± 0.12	0.57 ± 0.13	0.77*	0.76 ± 0.26	0.98
Succinate	0.07 ± 0.01	0.08 ± 0.00	1.18**	0.08 ± 0.02	0.85
Taurine	22.03 ± 1.97	29.58 ± 1.32	1.34***	28.41 ± 1.83	0.78***
Threonine	5.79 ± 0.82	3.37 ± 0.93	0.58**	4.41 ± 0.46	1.31**
Tyrosine	1.34 ± 0.28	1.24 ± 0.09	0.92	1.29 ± 0.16	1.04
Uracil	0.35 ± 0.03	0.31 ± 0.04	0.89	0.34 ± 0.05	1.02
Valine	5.65 ± 0.76	2.90 ± 0.25	0.51***	3.49 ± 0.56	1.62***

Day 7 Metabolites	Control – 7d			Temp – 7d				GSH – 7d			
	Mean	±	SD	Mean	±	SD	FC	Mean	±	SD	FC
Acetate	0.59	±	0.09	0.47	±	0.05	0.79*	0.55	±	0.03	1.08
Alanine	2.79	±	1.06	4.13	±	2.23	1.48	2.08	±	0.34	1.34
Arginine	2.43	±	0.40	1.95	±	0.41	0.80	1.81	±	0.24	1.34*
Aspartate	2.62	±	0.30	2.47	±	0.36	0.94	2.26	±	0.09	1.16*
Betaine	0.83	±	0.06	0.80	±	0.51	0.97	0.77	±	0.06	1.08
Choline	0.84	±	0.16	0.73	±	0.13	0.87	0.85	±	0.16	0.99
Citrate	0.34	±	0.04	0.30	±	0.21	0.89	0.37	±	0.08	0.91
Creatine	1.48	±	0.60	1.62	±	0.72	1.09	1.49	±	0.44	0.99
Dimethylamine	0.11	±	0.03	0.10	±	0.03	0.93	0.12	±	0.03	0.96
Formate	0.13	±	0.02	0.17	±	0.07	1.28	0.16	±	0.02	0.84
Fumarate	0.25	±	0.02	0.26	±	0.17	1.03	0.25	±	0.04	0.98
Glucose	1.97	±	0.54	2.13	±	0.22	1.08	1.78	±	0.60	1.11
Glutamate	9.36	±	0.35	8.46	±	0.39	0.90**	9.53	±	0.68	0.98
Glutamine	3.30	±	0.54	2.85	±	0.41	0.86	3.52	±	0.38	0.94
Glutarate	0.56	±	0.03	0.50	±	0.31	0.90	0.49	±	0.10	1.13
Glutathione	0.65	±	0.17	0.32	±	0.22	0.49*	0.76	±	0.22	0.85
Glycerol	1.41	±	0.38	1.69	±	0.59	1.20	1.32	±	0.26	1.07
Glycine	5.25	±	0.47	6.18	±	1.87	1.18	5.22	±	0.43	1.00
Isoleucine	1.30	±	0.52	1.63	±	0.68	1.25	1.54	±	0.39	0.85
Lactate	1.87	±	0.31	2.08	±	0.51	1.11	1.91	±	0.46	0.98
Leucine	2.91	±	0.56	3.02	±	0.48	1.04	3.06	±	0.33	0.95
Lysine	2.89	±	0.44	3.61	±	0.46	1.25*	3.20	±	0.49	0.90
Methionine	0.50	±	0.24	0.47	±	0.13	0.93	0.49	±	0.09	1.03
Myo-Inositol	3.75	±	0.94	4.52	±	2.68	1.20	4.11	±	0.75	0.91

O-Phosphocholine	0.22	±	0.06	0.39	±	0.22	1.78	0.23	±	0.02	0.96
O-Phosphoethanolamine	1.23	±	0.42	1.32	±	0.30	1.07	1.17	±	0.34	1.05
Ornithine	1.11	±	0.34	1.25	±	0.24	1.13	1.22	±	0.24	0.91
Phenylalanine	1.76	±	0.25	1.76	±	0.60	1.00	1.77	±	0.31	0.99
Proline	2.24	±	0.27	2.00	±	0.28	0.89	2.20	±	0.29	1.02
Serine	7.38	±	0.37	6.99	±	1.23	0.95	7.36	±	0.33	1.00
sn-Glycero-3-phosphocholine	0.66	±	0.15	0.99	±	0.08	1.49**	0.64	±	0.23	1.04
Succinate	0.10	±	0.03	0.07	±	0.04	0.71	0.08	±	0.01	1.26
Taurine	28.23	±	1.17	25.65	±	1.85	0.91*	28.38	±	0.67	0.99
Threonine	4.55	±	0.31	4.47	±	1.11	0.98	4.53	±	0.62	1.00
Tyrosine	0.95	±	0.21	1.01	±	0.14	1.07	1.22	±	0.28	0.78
Uracil	0.29	±	0.07	0.32	±	0.10	1.10	0.29	±	0.06	1.00
Valine	3.17	±	0.65	3.33	±	0.65	1.05	3.27	±	0.20	0.97

Day 10 Metabolites	Control – 10d			Temp – 10d				GSH – 10d			
	Mean	±	SD	Mean	±	SD	FC	Mean	±	SD	FC
Acetate	0.55	±	0.09	0.58	±	0.06	1.05	0.51	±	0.05	1.08
Alanine	2.09	±	0.40	2.06	±	0.48	0.99	2.25	±	0.41	0.93
Arginine	1.78	±	0.12	2.02	±	0.60	1.14	2.19	±	0.76	0.81
Aspartate	2.42	±	0.21	2.47	±	0.28	1.02	2.37	±	0.16	1.02
Betaine	0.81	±	0.11	0.67	±	0.09	0.83*	0.81	±	0.14	1.01
Choline	0.96	±	0.14	0.87	±	0.16	0.91	0.82	±	0.09	1.16
Citrate	0.40	±	0.15	0.32	±	0.13	0.80	0.29	±	0.15	1.37
Creatine	1.62	±	0.26	1.83	±	0.70	1.13	1.25	±	0.24	1.30*
Dimethylamine	0.12	±	0.02	0.10	±	0.03	0.88	0.11	±	0.02	1.10
Formate	0.15	±	0.04	0.14	±	0.05	0.96	0.12	±	0.04	1.22
Fumarate	0.24	±	0.05	0.21	±	0.06	0.90	0.21	±	0.07	1.14
Glucose	1.99	±	0.35	2.38	±	0.35	1.19	1.91	±	0.72	1.05
Glutamate	9.72	±	1.08	8.86	±	0.79	0.91	9.28	±	1.93	1.05
Glutamine	3.67	±	0.22	3.45	±	0.56	0.94	3.27	±	0.96	1.12
Glutarate	0.44	±	0.09	0.41	±	0.13	0.94	0.42	±	0.14	1.06
Glutathione	0.71	±	0.29	0.67	±	0.24	0.95	0.70	±	0.23	1.01
Glycerol	1.20	±	0.10	1.34	±	0.32	1.11	1.42	±	0.34	0.85
Glycine	4.74	±	0.97	6.02	±	1.34	1.27	6.25	±	2.44	0.76
Isoleucine	1.94	±	0.63	2.12	±	0.66	1.09	2.10	±	0.79	0.92
Lactate	1.70	±	0.22	2.30	±	1.00	1.35	1.69	±	0.61	1.01
Leucine	3.09	±	0.36	2.70	±	0.17	0.87*	3.37	±	0.33	0.92
Lysine	3.36	±	0.36	2.96	±	0.47	0.88	3.43	±	0.71	0.98
Methionine	0.79	±	0.24	0.87	±	0.27	1.10	0.75	±	0.10	1.05
Myo-Inositol	3.52	±	0.71	2.59	±	0.69	0.74*	3.59	±	1.18	0.98

O-Phosphocholine	0.20	±	0.04	0.32	±	0.08	1.58*	0.17	±	0.06	1.21
O-Phosphoethanolamine	1.22	±	0.36	1.09	±	0.36	0.89	0.83	±	0.28	1.48
Ornithine	1.17	±	0.16	1.64	±	0.28	1.40*	1.40	±	0.28	0.84
Phenylalanine	2.16	±	0.29	2.19	±	0.16	1.01	2.27	±	0.41	0.95
Proline	2.29	±	0.21	3.12	±	0.19	1.36***	2.34	±	0.57	0.98
Serine	7.00	±	0.73	8.32	±	0.49	1.19**	7.91	±	0.85	0.89
sn-Glycero-3-phosphocholine	1.10	±	0.74	0.87	±	0.27	0.80	0.91	±	0.28	1.21
Succinate	0.08	±	0.02	0.06	±	0.02	0.74	0.06	±	0.02	1.30
Taurine	27.17	±	1.91	23.18	±	2.69	0.85*	23.89	±	1.84	1.14*
Threonine	3.66	±	1.31	5.02	±	1.25	1.37	5.26	±	1.03	0.70*
Tyrosine	1.59	±	0.16	1.25	±	0.28	0.79*	1.39	±	0.41	1.14
Uracil	0.31	±	0.04	0.33	±	0.05	1.05	0.33	±	0.09	0.93
Valine	4.04	±	0.89	4.67	±	0.83	1.16	4.15	±	0.82	0.97

Day 14 Metabolites	Control – 14d			Temp – 14d				GSH – 14d			
	Mean	±	SD	Mean	±	SD	FC	Mean	±	SD	FC
Acetate	0.54	±	0.10	0.53	±	0.05	0.97	0.55	±	0.13	0.98
Alanine	2.49	±	0.48	2.31	±	0.52	0.93	2.93	±	0.84	0.85
Arginine	2.14	±	0.70	2.27	±	0.60	1.06	2.11	±	0.24	1.01
Aspartate	2.18	±	0.25	2.67	±	0.19	1.23**	2.35	±	0.12	0.93
Betaine	0.79	±	0.08	0.70	±	0.08	0.88	0.77	±	0.03	1.03
Choline	0.94	±	0.30	0.76	±	0.14	0.81	0.96	±	0.33	0.98
Citrate	0.36	±	0.16	0.41	±	0.06	1.14	0.32	±	0.12	1.12
Creatine	2.16	±	0.81	1.51	±	0.11	0.70	1.99	±	0.82	1.09
Dimethylamine	0.13	±	0.05	0.11	±	0.01	0.87	0.11	±	0.02	1.15
Formate	0.14	±	0.05	0.13	±	0.02	0.92	0.13	±	0.03	1.03
Fumarate	0.27	±	0.07	0.22	±	0.05	0.83	0.23	±	0.07	1.16
Glucose	1.86	±	0.22	2.23	±	0.12	1.20**	1.75	±	0.50	1.06
Glutamate	8.78	±	1.19	9.27	±	0.85	1.06	8.50	±	0.44	1.03
Glutamine	3.27	±	0.32	3.49	±	0.27	1.07	3.24	±	0.18	1.01
Glutarate	0.42	±	0.18	0.48	±	0.06	1.13	0.37	±	0.04	1.13
Glutathione	0.59	±	0.10	0.71	±	0.22	1.22	0.59	±	0.11	1.00
Glycerol	1.08	±	0.22	1.09	±	0.27	1.01	1.09	±	0.31	0.99
Glycine	5.15	±	1.31	5.47	±	0.69	1.06	4.85	±	0.68	1.06
Isoleucine	1.75	±	0.74	1.81	±	0.40	1.03	1.78	±	0.57	0.99
Lactate	1.52	±	0.30	2.00	±	0.15	1.31*	1.85	±	0.64	0.82
Leucine	2.73	±	0.45	3.11	±	0.39	1.14	3.41	±	0.62	0.80
Lysine	3.20	±	0.52	3.00	±	0.23	0.94	3.09	±	0.30	1.03
Methionine	0.90	±	0.27	0.57	±	0.29	0.63	0.72	±	0.15	1.25
Myo-Inositol	3.04	±	0.32	2.15	±	0.26	0.71**	2.58	±	0.32	1.18*

O-Phosphocholine	0.17	±	0.09	0.21	±	0.08	1.19	0.19	±	0.07	0.93
O-Phosphoethanolamine	1.02	±	0.27	0.60	±	0.19	0.58*	0.87	±	0.36	1.18
Ornithine	1.22	±	0.28	1.34	±	0.16	1.10	1.22	±	0.39	1.00
Phenylalanine	2.18	±	0.50	2.00	±	0.20	0.91	2.09	±	0.17	1.04
Proline	2.06	±	0.30	2.57	±	0.44	1.25*	2.15	±	0.40	0.96
Serine	7.07	±	0.80	7.87	±	0.46	1.11	6.30	±	2.06	1.12
sn-Glycero-3-phosphocholine	0.84	±	0.17	1.04	±	0.27	1.24	1.11	±	0.51	0.75
Succinate	0.07	±	0.02	0.06	±	0.02	0.75	0.06	±	0.01	1.29
Taurine	29.58	±	1.64	26.99	±	1.84	0.91*	29.87	±	1.95	0.99
Threonine	3.83	±	1.30	4.52	±	0.58	1.18	4.43	±	0.72	0.86
Tyrosine	1.16	±	0.36	1.34	±	0.37	1.16	1.05	±	0.37	1.11
Uracil	0.32	±	0.06	0.29	±	0.03	0.92	0.31	±	0.04	1.05
Valine	4.05	±	1.35	4.18	±	0.73	1.03	4.10	±	1.07	0.99

Table S2. Normalized concentration of metabolites in the liver.

Normalized concentrations are expressed as means ± standard deviation (SD).

Fold change = (treatment)/(control)

*, P < 0.05; **, P < 0.01; ***, P < 0.001

Day 4	Control – 4d			Temp – 4d				GSH – 4d			
Metabolites	Mean	±	SD	Mean	±	SD	FC	Mean	±	SD	FC
Acetate	0.41	±	0.10	0.33	±	0.05	0.816	0.30	±	0.04	0.74*
Alanine	6.15	±	0.86	5.72	±	0.24	0.929	6.06	±	2.19	0.985
Asparagine	0.80	±	0.20	0.85	±	0.23	1.062	0.76	±	0.08	0.948
Aspartate	3.94	±	0.32	3.35	±	0.25	0.85**	3.19	±	0.14	0.81**
Choline	1.66	±	0.39	2.41	±	1.04	1.455	2.82	±	0.82	1.71*
Creatine	2.13	±	0.78	2.69	±	0.50	1.263	2.15	±	0.29	1.008
Fumarate	0.17	±	0.05	0.23	±	0.04	1.347	0.19	±	0.01	1.103
Glucose	5.74	±	0.69	10.85	±	0.63	1.89***	6.75	±	0.95	1.175
Glutamate	11.86	±	0.91	10.04	±	1.02	0.85*	11.08	±	0.76	0.934
Glutamine	3.39	±	0.62	2.87	±	0.13	0.848	2.85	±	0.42	0.841
Glutathione	0.50	±	0.12	0.74	±	0.04	1.48**	0.74	±	0.03	1.5**
Glycerol	4.21	±	1.51	5.60	±	0.35	1.332	5.53	±	1.38	1.315
Glycine	2.17	±	0.14	2.02	±	0.07	0.93*	2.20	±	0.12	1.011
Histidine	1.15	±	0.29	0.81	±	0.09	0.7*	0.77	±	0.15	0.67*
Hypoxanthine	1.65	±	0.49	1.49	±	0.16	0.906	1.71	±	0.23	1.037
Inosine	0.57	±	0.16	0.79	±	0.24	1.375	0.49	±	0.11	0.848
Isoleucine	1.35	±	0.59	0.72	±	0.10	0.53*	0.76	±	0.10	0.56*
Leucine	3.49	±	1.55	2.00	±	0.37	0.57*	1.93	±	0.35	0.55*
Lysine	2.03	±	0.44	1.41	±	0.30	0.7*	1.41	±	0.28	0.7*

Malate	2.61	±	0.75	3.14	±	0.46	1.205	2.86	±	0.15	1.097
Malonate	0.29	±	0.04	0.37	±	0.08	1.281	0.33	±	0.05	1.136
Maltose	2.53	±	1.27	5.57	±	0.84	2.2**	3.35	±	0.32	1.322*
Methionine	1.06	±	0.42	0.65	±	0.12	0.61*	0.63	±	0.11	0.6
Myo-Inositol	0.60	±	0.20	0.76	±	0.16	1.267	0.71	±	0.03	1.177
N,N-Dimethylglycine	0.07	±	0.03	0.07	±	0.03	1.024	0.08	±	0.07	1.112*
O-Phosphocholine	0.12	±	0.05	0.15	±	0.04	1.294	0.16	±	0.05	1.369
Ornithine	0.72	±	0.07	0.52	±	0.03	0.73***	0.51	±	0.08	0.7**
Phenylalanine	1.14	±	0.64	0.58	±	0.13	0.51	0.59	±	0.16	0.519
Proline	2.11	±	0.23	1.60	±	0.22	0.76**	1.60	±	0.18	0.76**
Serine	3.65	±	0.52	3.20	±	0.42	0.876	3.16	±	0.43	0.867
sn-Glycero-3-phosphocholine	0.55	±	0.05	0.63	±	0.09	1.148	0.76	±	0.24	1.389
Succinate	0.13	±	0.11	0.30	±	0.07	2.3*	0.28	±	0.06	2.11*
Taurine	22.58	±	1.48	21.32	±	0.55	0.944	27.45	±	2.18	1.22**
Threonine	2.04	±	0.55	1.50	±	0.18	0.737	1.39	±	0.19	0.68*
Tryptophan	0.33	±	0.20	0.24	±	0.04	0.728	0.19	±	0.05	0.577
Tyrosine	1.28	±	0.53	0.72	±	0.16	0.56*	0.75	±	0.15	0.58*
Valine	2.66	±	1.06	1.56	±	0.24	0.59*	1.57	±	0.24	0.59*

Day 7 Metabolites	Control – 7d			Temp – 7d				GSH – 7d			
	Mean	±	SD	Mean	±	SD	FC	Mean	±	SD	FC
Acetate	0.31	±	0.06	0.24	±	0.04	0.77*	0.30	±	0.05	0.97
Alanine	3.81	±	0.47	4.33	±	1.18	1.14	3.82	±	0.41	1.00
Asparagine	1.44	±	0.39	1.08	±	0.15	0.75	2.49	±	1.70	1.73
Aspartate	3.42	±	0.26	2.75	±	0.25	0.80**	3.14	±	0.35	0.92
Choline	2.69	±	0.34	2.61	±	0.71	0.97	2.00	±	0.41	0.74*
Creatine	3.40	±	0.51	3.50	±	0.75	1.03	2.95	±	0.82	0.87
Fumarate	0.19	±	0.03	0.23	±	0.05	1.23	0.19	±	0.08	1.01
Glucose	11.71	±	1.01	14.16	±	1.78	1.21*	11.66	±	1.71	1.00
Glutamate	14.24	±	0.88	14.64	±	1.17	1.03	13.62	±	2.27	0.96
Glutamine	3.05	±	0.51	2.34	±	0.70	0.77	3.45	±	1.05	1.13
Glutathione	0.56	±	0.08	0.87	±	0.08	1.55***	0.69	±	0.09	1.23*
Glycerol	4.27	±	0.31	4.85	±	0.73	1.14	3.15	±	1.01	0.74*
Glycine	1.83	±	0.36	1.57	±	0.42	0.86	1.79	±	0.25	0.98
Histidine	0.66	±	0.05	0.62	±	0.12	0.93	0.74	±	0.10	1.11
Hypoxanthine	1.82	±	0.38	1.09	±	0.22	0.60**	0.78	±	0.16	0.43***
Inosine	0.40	±	0.14	0.53	±	0.29	1.31	0.67	±	0.21	1.65*
Isoleucine	0.53	±	0.06	0.42	±	0.18	0.78	0.60	±	0.35	1.12
Leucine	1.15	±	0.16	0.97	±	0.45	0.84	1.58	±	1.04	1.38
Lysine	1.53	±	0.28	1.46	±	0.36	0.96	1.64	±	0.59	1.08
Malate	3.02	±	0.29	2.91	±	0.46	0.96	2.77	±	0.70	0.92
Malonate	0.31	±	0.06	0.34	±	0.05	1.11	0.26	±	0.03	0.86
Maltose	0.19	±	0.06	0.89	±	0.14	4.73***	0.54	±	0.12	2.83***
Methionine	0.37	±	0.06	0.34	±	0.13	0.92	0.55	±	0.34	1.46
Myo-Inositol	1.52	±	0.12	2.39	±	0.55	1.57**	1.47	±	0.32	0.97

N,N-Dimethylglycine	0.06	±	0.02	0.10	±	0.05	1.64	0.06	±	0.01	1.04
Ornithine	0.39	±	0.07	0.29	±	0.07	0.74*	0.33	±	0.06	0.85
O-Phosphocholine	0.13	±	0.02	0.17	±	0.03	1.33*	0.12	±	0.02	0.91
Phenylalanine	0.33	±	0.07	0.41	±	0.18	1.25	0.57	±	0.47	1.73
Proline	1.57	±	0.15	1.34	±	0.25	0.86	1.64	±	0.27	1.04
Serine	2.34	±	0.26	2.57	±	0.61	1.10	2.62	±	0.71	1.12
sn-Glycero-3-phosphocholine	0.61	±	0.09	0.54	±	0.12	0.89	0.37	±	0.06	0.60***
Succinate	0.35	±	0.08	0.52	±	0.26	1.49	0.35	±	0.24	1.02
Taurine	27.34	±	0.98	24.66	±	2.52	0.90*	28.63	±	2.11	1.05
Threonine	1.00	±	0.17	1.00	±	0.34	1.00	1.06	±	0.43	1.06
Tryptophan	0.12	±	0.05	0.18	±	0.09	1.42	0.19	±	0.12	1.58
Tyrosine	0.47	±	0.04	0.39	±	0.22	0.83	0.54	±	0.29	1.15
Valine	0.97	±	0.13	0.90	±	0.40	0.93	1.17	±	0.65	1.21

Day 10 Metabolites	Control – 10d			Temp – 10d				GSH – 10d			
	Mean	±	SD	Mean	±	SD	FC	Mean	±	SD	FC
Acetate	0.45	±	0.09	0.32	±	0.05	0.72*	0.45	±	0.08	1.01
Alanine	4.87	±	1.58	4.37	±	0.71	0.90	3.53	±	1.04	0.72
Asparagine	1.33	±	0.48	1.79	±	0.52	1.35	1.79	±	0.28	1.35
Aspartate	2.49	±	0.48	3.07	±	0.47	1.24	2.75	±	0.57	1.11
Choline	1.85	±	0.10	1.87	±	0.29	1.01	2.66	±	0.26	1.44***
Creatine	4.74	±	1.11	3.42	±	0.88	0.72*	4.05	±	0.63	0.86
Fumarate	0.22	±	0.03	0.24	±	0.05	1.08	0.16	±	0.04	0.71*
Glucose	10.58	±	2.24	11.61	±	2.52	1.10	10.76	±	2.17	1.02
Glutamate	14.11	±	0.43	14.19	±	0.70	1.01	15.25	±	1.27	1.08
Glutamine	2.94	±	0.33	2.70	±	0.47	0.92	3.11	±	0.75	1.06
Glutathione	0.62	±	0.05	0.95	±	0.11	1.52***	0.75	±	0.05	1.21**
Glycerol	4.87	±	0.75	4.52	±	0.91	0.93	3.19	±	0.68	0.66**
Glycine	1.31	±	0.18	1.32	±	0.16	1.00	1.57	±	0.15	1.19*
Histidine	0.57	±	0.06	0.67	±	0.10	1.18	0.63	±	0.21	1.11
Hypoxanthine	1.20	±	0.18	1.12	±	0.25	0.93	0.88	±	0.31	0.74
Inosine	0.80	±	0.29	0.72	±	0.26	0.90	0.67	±	0.06	0.83
Isoleucine	0.39	±	0.12	0.46	±	0.10	1.18	0.47	±	0.27	1.20
Leucine	0.97	±	0.32	1.13	±	0.33	1.16	1.33	±	1.06	1.37
Lysine	1.03	±	0.24	1.42	±	0.31	1.38*	1.27	±	0.67	1.23
Malate	2.99	±	0.37	3.44	±	0.58	1.15	2.45	±	0.22	0.82*
Malonate	0.28	±	0.07	0.30	±	0.05	1.08	0.32	±	0.06	1.15
Maltose	1.11	±	0.58	2.51	±	1.38	2.25*	0.25	±	0.10	0.23**
Methionine	0.39	±	0.13	0.47	±	0.11	1.18	0.51	±	0.33	1.30
Myo-Inositol	1.09	±	0.21	0.74	±	0.06	0.96**	1.10	±	0.39	0.88

N,N-Dimethylglycine	0.04	±	0.02	0.08	±	0.02	2.07**	0.04	±	0.01	0.98
Ornithine	0.31	±	0.06	0.35	±	0.05	1.31	0.36	±	0.10	1.51
O-phosphocholine	0.13	±	0.02	0.12	±	0.02	0.96	0.13	±	0.02	1.02
Phenylalanine	0.36	±	0.07	0.47	±	0.13	0.97	0.54	±	0.35	0.99
Proline	1.34	±	0.16	1.31	±	0.16	1.13*	1.33	±	0.15	1.15
Serine	1.80	±	0.18	2.03	±	0.16	0.90	2.07	±	0.24	0.77
sn-Glycero-3-phosphocholine	0.41	±	0.05	0.40	±	0.03	1.70	0.36	±	0.10	1.09
Succinate	0.49	±	0.08	0.44	±	0.14	0.89**	0.37	±	0.17	1.02
Taurine	30.52	±	1.69	27.26	±	1.02	1.31	31.21	±	2.48	1.16
Threonine	0.82	±	0.22	1.07	±	0.24	1.30	0.95	±	0.41	1.32
Tryptophan	0.15	±	0.04	0.20	±	0.04	1.15	0.20	±	0.06	1.22
Tyrosine	0.37	±	0.10	0.42	±	0.11	1.20	0.45	±	0.31	0.65
Valine	0.77	±	0.29	1.02	±	0.32	0.68	0.98	±	0.69	1.0

Day 14 Metabolites	Control – 14d			Temp – 14d				GSH – 14d			
	Mean	±	SD	Mean	±	SD	FC	Mean	±	SD	FC
Acetate	0.34	±	0.06	0.40	±	0.07	1.19	0.32	±	0.04	0.96
Alanine	5.58	±	3.20	3.45	±	0.93	0.62	3.54	±	0.60	0.63
Asparagine	1.44	±	0.30	1.22	±	0.37	0.84	1.16	±	0.48	0.81
Aspartate	1.81	±	0.20	3.07	±	0.33	1.70***	1.74	±	0.22	0.96
Choline	2.62	±	0.48	2.80	±	0.22	1.07	2.96	±	0.73	1.13
Creatine	4.77	±	0.57	3.98	±	1.44	0.84	3.56	±	1.05	0.75*
Fumarate	0.19	±	0.03	0.19	±	0.04	0.98	0.21	±	0.04	1.09
Glucose	8.94	±	1.94	12.98	±	2.18	1.45*	9.31	±	0.82	1.04
Glutamate	16.13	±	1.01	13.84	±	1.43	0.86*	13.99	±	1.48	0.87*
Glutamine	2.80	±	0.50	2.37	±	0.59	0.84	2.30	±	0.12	0.82*
Glutathione	0.44	±	0.04	0.57	±	0.13	1.31*	0.52	±	0.12	1.18
Glycerol	3.68	±	1.33	3.09	±	0.19	0.84	3.27	±	0.45	0.89
Glycine	1.02	±	0.26	1.59	±	0.35	1.55*	0.94	±	0.11	0.92
Histidine	0.50	±	0.07	0.64	±	0.10	1.29*	0.43	±	0.03	0.87
Hypoxanthine	0.78	±	0.12	1.04	±	0.26	1.34	0.76	±	0.14	0.98
Inosine	0.70	±	0.22	0.77	±	0.24	1.10	0.68	±	0.20	0.98
Isoleucine	0.31	±	0.13	0.48	±	0.04	1.56*	0.23	±	0.03	0.76
Leucine	0.78	±	0.08	0.94	±	0.31	1.21	0.47	±	0.09	0.61***
Lysine	0.71	±	0.13	1.43	±	0.27	2.02***	0.63	±	0.06	0.88
Malate	2.46	±	0.32	2.58	±	0.28	1.05	2.72	±	0.53	1.11
Malonate	0.27	±	0.02	0.32	±	0.05	1.19*	0.28	±	0.02	1.03
Maltose	0.25	±	0.07	0.26	±	0.06	1.06	0.18	±	0.03	0.74
Methionine	0.29	±	0.13	0.57	±	0.10	1.93**	0.23	±	0.04	0.78
Myo-Inositol	1.07	±	0.37	0.90	±	0.25	0.84	1.48	±	0.22	1.38*

N,N-Dimethylglycine	0.29 ± 0.13	0.55 ± 0.10	1.93**	0.22 ± 0.04	0.78
Ornithine	0.25 ± 0.05	0.29 ± 0.06	1.17	0.19 ± 0.03	0.79
O-Phosphocholine	0.14 ± 0.02	0.24 ± 0.20	1.75	0.13 ± 0.01	0.92
Phenylalanine	0.29 ± 0.15	0.55 ± 0.12	1.87*	0.22 ± 0.03	0.75
Proline	1.02 ± 0.14	1.61 ± 0.29	1.58**	1.15 ± 0.21	1.13
Serine	1.57 ± 0.38	2.51 ± 0.26	1.60**	1.46 ± 0.25	0.93
sn-Glycero-3-phosphocholine	0.35 ± 0.03	0.39 ± 0.06	1.12	0.31 ± 0.05	0.88
Succinate	0.62 ± 0.11	0.45 ± 0.16	0.73	0.80 ± 0.14	1.29*
Taurine	34.44 ± 1.98	29.64 ± 1.59	0.86**	40.94 ± 2.18	1.19**
Threonine	0.53 ± 0.07	1.10 ± 0.11	2.07***	0.51 ± 0.05	0.96
Tryptophan	0.11 ± 0.05	0.20 ± 0.04	1.79**	0.11 ± 0.05	1.01
Tyrosine	0.28 ± 0.17	0.47 ± 0.13	1.70	0.22 ± 0.05	0.78
Valine	0.67 ± 0.18	1.11 ± 0.13	1.66**	0.43 ± 0.07	0.65*

Figure S1. Changes in the biochemical parameters of serum in olive flounder after thermal stress.

CON, control group; Temp, Temp group; GSH, GSH group; d, day

AST, aspartate aminotransferase; ALT, aminotransferase; BUN, blood urea nitrogen; TP, total protein; ALP, alkaline phosphatase; TCHOL, total cholesterol

*, $P < 0.05$

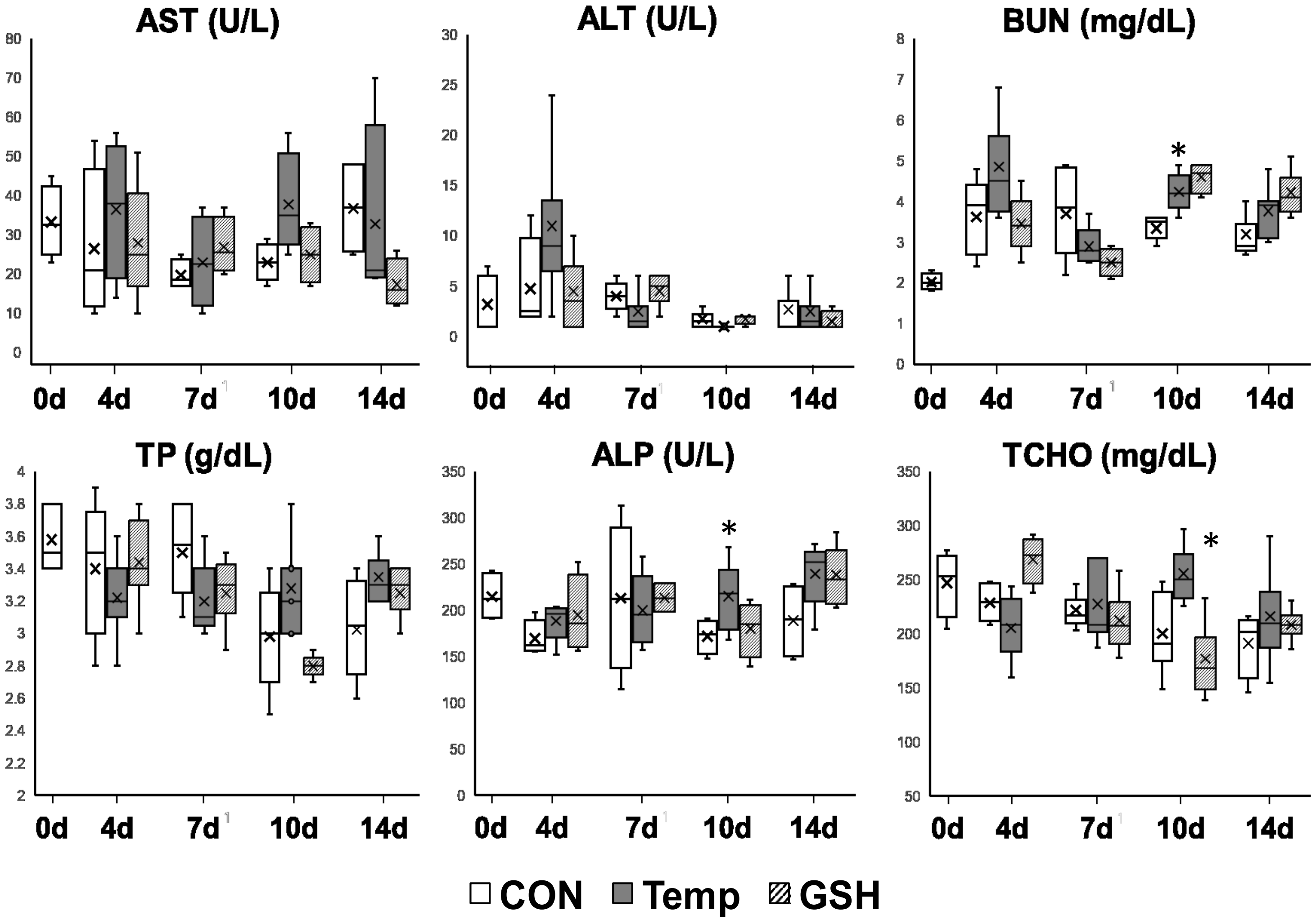

Supplement: Supplementary file 1 [file metabolites-10-00003-s001.pdf]
